# Measurement, determinants and outcomes of maternal care satisfaction in Nigeria: a systematic review

**DOI:** 10.1136/bmjph-2024-001278

**Published:** 2025-02-11

**Authors:** Beatrice Wuraola Ope, Tavleen Wasan, Jane E Hirst, Edward Mullins, Robyn Norton, Margaret Peden

**Affiliations:** 1 Imperial College London Faculty of Medicine, London, UK; 2 Imperial College London, The George Institute for Global Health UK, London, UK; 3 University of Oxford Nuffield Department of Women's & Reproductive Health, Oxford, Oxfordshire, UK; 4 Imperial College London Department of Metabolism Digestion and Reproduction, London, UK; 5 The George Institute for Global Health, Sydney, New South Wales, Australia

**Keywords:** Public Health, Epidemiologic Research Design, Demography

## Abstract

**Introduction:**

Maternal mortality rates are unacceptably high in Nigeria. Understanding women’s satisfaction with the maternity health system is fundamental, as perceived quality of care is a determinant of service utilisation and improved birth outcomes.

**Objectives:**

This systematic review aims to explore patients’ satisfaction with maternal healthcare in Nigeria, examining the measurement, determinants, and outcomes of satisfaction.

**Design:**

A systematic review following the Preferred Reporting Items for Systematic Reviews and Meta-Analyses 2020 guidelines.

**Data sources:**

Searches were conducted in Embase, Maternity and Infant Care, Global Health, Ovid, Africa Journals Online, Cochrane Central Register of Controlled Trials, MEDLINE, Web of Science, Cumulative Index to Nursing and Allied Health Literature (CINAHL), Scopus and Google Scholar, alongside citation searches of relevant studies.

**Eligibility criteria:**

Original studies assessing patient satisfaction with maternal health services in Nigeria were included. There were no restrictions on study design types. Studies were excluded if they did not clearly define how satisfaction was measured or did not focus on the maternal health service domains under review (ie, antenatal, delivery, and/or postnatal care).

**Data extraction and synthesis:**

Key information relevant to this study was extracted into an Excel spreadsheet and narratively synthesised. The Quality Assessment for Diverse Studies tool was used to appraise the quality of the selected literature.

**Results:**

Maternal care satisfaction (MCS) ratings are high in Nigeria, but this may either indicate genuine positive experiences or be influenced by measurement-related biases. Several factors determine women’s satisfaction with maternity services. Key factors influencing MCS in Nigeria are positive client-provider relationships, a favourable hospital environment with easy accessibility and affordable care costs. While our study demonstrated a correlation between MCS and socioeconomic and demographic factors, there is no complete consensus within the literature about this correlation. Furthermore, patient satisfaction was associated with women’s future health-seeking behaviour and willingness to recommend care to others.

**Conclusion:**

Understanding the multifaceted nature of MCS determinants and outcomes can better equip us to provide the support and care that mothers need to thrive. The findings from this study can inform policy, improve health responsiveness and ensure that women are provided with satisfactory and patient-centred maternity care, hence leading to a decline in poor pregnancy outcomes in Nigeria. It also highlights the need for robust methodologies that accurately measure women’s experiences, which is essential for enhancing the quality of maternal health services.

**PROSPERO registration number:**

A protocol was developed for this study and published on PROSPERO, the International Prospective Register of Systematic Reviews (CRD42023414771).

**WHAT IS ALREADY KNOWN ON THIS TOPIC**

- The satisfaction of mothers with maternal care in Nigeria is crucial for the health and well-being of both the mother and the child.

**WHAT THIS STUDY ADDS**

- This systematic review study provides insights into the measurement, determinants and outcomes of maternal care satisfaction (MCS) in Nigeria. It synthesises findings from various studies to provide a comprehensive overview of MCS across Nigeria, addressing the absence of a single nationwide study on this topic. By integrating data from diverse geographic areas, it offers a broader understanding of maternal care quality and satisfaction in the country.

**HOW THIS STUDY MIGHT AFFECT RESEARCH, PRACTICE OR POLICY**

- The findings of this study can guide healthcare providers in developing targeted, evidence-based interventions to enhance maternal care quality and patient satisfaction in Nigeria and similar settings. It also highlights the need for robust methodologies that accurately measure women’s experiences, which is essential for enhancing the quality of maternal health services.

## Introduction

 Approximately 800 women die daily from pregnancy-related complications worldwide, equating to one death every 2 min.^1^ The global maternal mortality ratio (MMR) decreased by 34% (from 342 to 223 deaths per 100 000 live births) between 2000 and 2020, but disparities across geographies persist.^2^ In 2020, MMR in low-income countries (LICs) was 430 per 100 000 live births, compared with 12 in high-income countries (HICs).^1^ About 94% of the 295 000 deaths in 2017 occurred in low-resource settings, with southeast Asia and sub-Saharan Africa contributing over 80% (254 000).^3^ Sub-Saharan Africa alone represented 70% of global maternal mortality in 2020.^2^ These discrepancies highlight unequal access to quality health services.^1^

Nigeria, with a population of around 200 million, bears 28.5% of global maternal mortality.^2 4^ The maternal mortality rate in the country rose by 14% from 2017 to 2020 (from 917 to 1047 deaths per 100 000 live births).^5^ Despite the WHO’s recommendation of skilled birth attendants, less than 40% of deliveries in Nigeria occur in formal health facilities where women can access skilled birth attendants.^1 6^ The majority of women opt for home or traditional birth centre deliveries.^6^ Poor quality maternity healthcare, spanning antenatal, delivery and postnatal care significantly contribute to adverse pregnancy outcomes.^1 7^ The WHO emphasises the importance of routine assessment of women’s satisfaction for enhancing care quality and service utilisation.^8^

Patient satisfaction refers to how contented or pleased a patient is with the healthcare received.^9 10^ It is subjective as it is shaped by their expectations of care.^11^ It does not matter whether the patient is right or wrong, all that matters is how the patient feels.^12^ Patient satisfaction serves as a valid metric for assessing maternity care and evaluating success-guiding improvements in care quality.^9^,^12^ Countries such as France in 1996 mandated hospitals to report patient satisfaction, and since 2005, Germany has included satisfaction assessments in quality management reports.^13^ The UK also conduct routine maternity surveys to assess the experiences of pregnant women with the health system, thereby facilitating an evaluation of National Health Service performance and identifying areas requiring enhancement.^14^ Therefore, patient satisfaction is pivotal in enhancing healthcare industry performance.

Maternal care satisfaction (MCS) is vital for both maternal and child well-being, impacting health service utilisation.^15 16^ Perceived healthcare quality influences women’s behaviour, affecting adherence to care, trust in the health system and follow-up appointments.^12 13^ Support and respect contribute to trust in care providers, increasing compliance.^10 17^ Conversely, dissatisfaction may lead to reliance on traditional care and a last-resort approach to formal health institutions.^16^,^18^

While many studies on MCS in Nigeria focus on specific states or regions, national-level research is limited.^18^,^21^ The only national study on MCS in Nigeria concentrated solely on intrapartum care, overlooking antenatal and postnatal care.^22^ Antenatal and postnatal care are important components of maternal healthcare and should not be left out in patient satisfaction research. For example, a Lancet study revealed that haemorrhage, which mainly occurs during the postpartum period, is the leading direct cause of maternal mortality in the world.^23^ Similarly, timely antenatal care (ANC) initiation aids in managing pregnancy complications and reducing maternal mortality.^16 19 21^ If women are unsatisfied with ANC, they will be less likely to opt for institutional delivery.^16^

Therefore, this study aims to fill the gap in the literature by exploring the factors affecting women’s satisfaction with maternal healthcare services in Nigeria. With maternal mortality rates alarmingly high and showing a concerning trend of increase in Nigeria, it is crucial to ascertain whether MCS play a contributing role, which is essential for informing national and regional strategies aimed at reducing maternal mortality ratio (MMR). By investigating the measurements, determinants and outcomes of MCS, this study seeks to provide new evidence to help shape policy development and the design of maternal healthcare programmes.

## Methods

### Eligibility criteria

An initial literature review was conducted to help develop a protocol, inclusion criteria and research questions. A protocol was then developed for this study and published on PROSPERO, the International Prospective Register of Systematic Reviews (CRD42023414771). This systematic review considered original studies that assessed patient satisfaction with maternal health services in Nigeria. There was no restriction on the study design type eligible for inclusion to include both qualitative and quantitative studies. We excluded studies where women were pregnant more than one year ago to reduce recall bias, studies that did not clearly define how satisfaction was measured, systematic review studies and studies that did not focus on the maternal health service domains under review (ie, antenatal, delivery and/or postnatal care). The population, intervention, comparator, outcomes, timing and setting guide informed the research question and eligibility criteria (table 1).^24^

**Table 1:** Eligibility criteria using PICOT

PICOT element	Description
Population	Women of reproductive age (15–49 years) who have used maternal healthcare in Nigeria and reported their satisfaction with the services received.
Intervention or exposure	Utilisation of maternal healthcare services such as antenatal care (AC), intrapartum care (IC), and/or postpartum care (PC) in Nigeria.
Comparator	Studies with any comparator group and those with no comparator group were both included.
Outcome	Primary outcome: Patients’ satisfaction with maternal health services (AC, IC and PC) in Nigeria.Secondary outcome: Determinants and measurement of maternal care satisfaction in Nigeria.Tertiary outcome: Outcomes of women’s satisfaction with maternal services. Satisfaction outcomes in this study refer to (i) association of satisfaction with continued use (return to the facility) and (ii) association of satisfaction with patient referrals (women’s willingness to recommend care to others).
Timing	The search was not limited by date to reach as many publications as possible.

PICOT, population, intervention, comparator, outcomes, timing.

### Search strategy

A search strategy was constructed based on the literature review with the assistance of a librarian. Comprehensive searches were conducted across multiple databases, including Embase, Maternity and Infant and Care, Global Health, Ovid, Africa Journals Online, Cochrane Central Register of Controlled Trials (CENTRAL), MEDLINE, Web of Science, Cumulative Index to Nursing and Allied Health Literature (CINAHL), Scopus and Google Scholar focusing on studies examining women’s satisfaction with maternal health services in Nigeria.

We also searched through the reference lists of retrieved studies for literature not identified by the search. The search was not limited by date or language to reach as many papers as possible. The search strategy was initially developed in MEDLINE using MeSH terms and keywords related to the topic (see online supplemental table S1.1) and then adapted for use across other databases, following a consistent approach that combined population, intervention and outcome terms. In databases where combined searches were not feasible, individual searches were carried out for relevant indicators of MCS with a focus on studies conducted in Nigeria. Synonyms, homonyms and variations of keywords were employed to capture all relevant studies. The final search was completed on 31 October 2023.

### Selection process

Following a comprehensive search across different databases, the identified articles were imported into Covidence, a screening and data extraction tool, for deduplication and screening.^25^ Two reviewers (BWO and TW) independently screened the title and abstract of the articles based on the eligibility criteria. Thereafter, full-text articles were identified and independently evaluated by the two reviewers. Disagreements were resolved on study selection and data extraction by consensus when and where necessary.

### Data collection process

Study information such as author, year of publication, study location, baseline population characteristics, women’s satisfaction level measurement tools and factors associated with satisfaction were extracted into an Excel spreadsheet. Authors were contacted if any information provided was ambiguous.

### Study risk of bias assessment

The Quality Assessment for Diverse Studies (QuADS) tool, a 13-item checklist, was used to appraise the quality of the selected literature.^26^ The QuADS quality assessment tool was developed for use in systematic reviews that include mixed-methods research.^26^ The quality of the included studies was assessed by the two reviewers, BWO and TW, and if any disagreement arose, a consensus was reached by discussion.

### Synthesis method

It was planned to use meta-analysis to estimate the pooled rate of maternal satisfaction through a random-effect model. However, this was impeded by the heterogeneity of tools and instruments used across studies to measure MCS. Therefore, the study findings were narratively synthesised. Satisfaction scores were derived from specific questions asked in these studies to assess participants’ overall satisfaction with the maternal healthcare they received, such as, ‘Overall, were you satisfied with the care received?’ (online supplemental table S2.2). This information was extracted directly from the respective studies. In cases where direct scores were not available, satisfaction rates were calculated by combining responses from related subquestions or subgroups within the participants’ responses (online supplemental table S2.2).

Information from each study was structured into a table and then summarised using words and text. Patient satisfaction was synthesised using a 5-point Likert-type scale and converted to percentages with 100% representing ‘very high’, 80% representing ‘high’, 60% representing ‘moderately satisfied’, 40% representing ‘low’ and 0% representing ‘no satisfaction’.^27^ The determinants of MCS were categorised into themes based on research evidence.^10^,^13^ The determinants were further classified into factors that are linked with satisfaction and dissatisfaction with care.

### Patient and public statement

There was no patient or public involvement in this research.

## Results

### Study selection

The study followed the Preferred Reporting Items for Systematic Reviews and Meta-Analyses (PRISMA) 2020 guideline.^28^ The search yielded 3117 studies from different databases—628 from MEDLINE, 1298 from Embase, 215 from Maternity and Infant and Care, 610 from Global Health, 202 from Web of Science, 83 from CINAHL, 25 from Scopus and 56 from Google Search and searches of citations from published papers.

**Figure 1:**
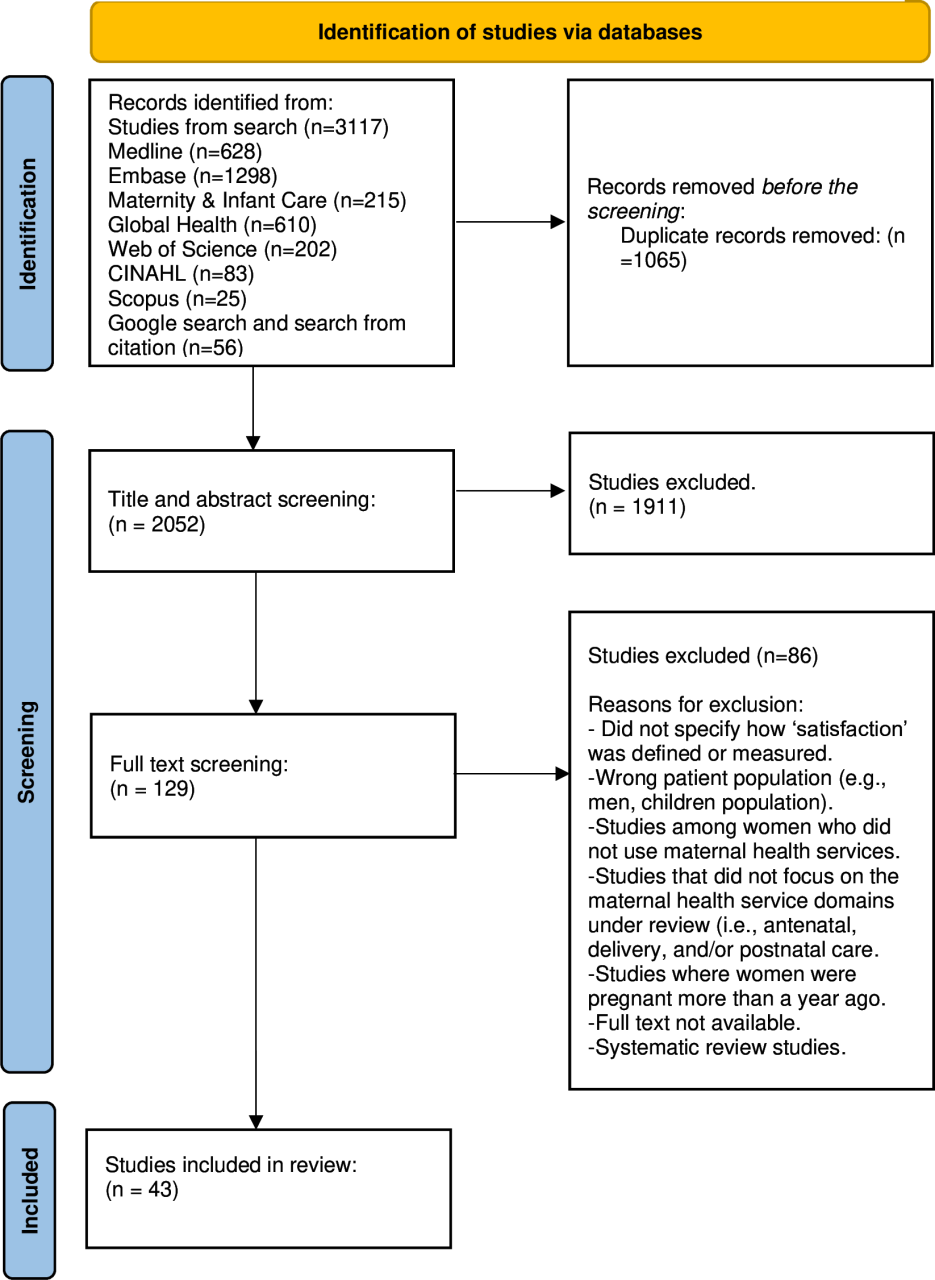
Preferred Reporting Items for Systematic Reviews and Meta-Analyses (PRISMA) flow diagram of the study. The search methodology followed established inclusion and exclusion criteria according to PRISMA guidelines, as detailed above. The initial search yielded a total of 3117 studies, with exclusions made for undefined satisfaction measures, incorrect populations or lack of focus on maternal health service domains (ie, antenatal, delivery and/or postnatal care). This process resulted in 43 studies being included in the systematic review. CINAHL, Cumulative Index to Nursing and Allied Health Literature.

After removing 1065 duplicates, 2052 papers were retained and screened by title and abstract. Subsequently, 1911 studies were excluded, and 129 articles were retained and screened by full text. After the full-text screening, 86 studies were excluded, and 43 papers were included in the review. Studies were excluded because they either did not specify how satisfaction was defined or measured, had only an abstract and not the full text, had a wrong patient population, were systematic review studies or did not focus on the maternal health service domains under review (ie, antenatal, delivery and/or postnatal care). The review process is presented in the PRISMA flow diagram in figure 1.

### Study characteristics

The 43 studies analysed in this research encompassed a wide range of sample sizes, spanning from nine to 2262 participants. Data collection methods included questionnaires, interviews and focus-group discussions. The studies considered various healthcare levels: 17 tertiary facilities, nine secondary facilities and 11 primary health facilities. The research focused on numerous maternal health services, with 26 studies on ANC, 12 on intrapartum (delivery) care, one on antenatal and postnatal care, and four on all types of maternal health services. For a comprehensive summary of each study’s characteristics, please refer to onlinesupplemental tables S1.2, S2.1  S2.2.

### Risk of bias in studies

Each paper was scored based on the QuADS checklist requirement and then aggregated to determine the quality of the study, with a maximum score of 39.^26^ For ease of interpretation, the scores were converted to percentages and were categorised based on a similar systematic review—studies scoring above 75% were considered ‘high quality’, 50–75% ‘good quality’, 25–50% ‘moderate quality’ and those scoring below 25% ‘poor quality’.^26^ Among the studies considered, one was assessed as high quality, 40 were assessed as good quality, and two were categorised as moderate quality (online supplemental table S2.3).

### Measurement of satisfaction

All included studies evaluated women’s satisfaction with the maternity care received, describing the measurement methods employed (See onlinesupplemental table S1.2 S2.2).

Of the 43 studies selected, 37 used quantitative research methods,^19^,^2127 29^ three used qualitative,^61^,^63^ and the other three used mixed methods.^64^,^66^

Among the quantitative studies, the majority, 27 studies, collected data using interviewer-administered questionnaires,^2021 29 30 33^,^40 42 43 45^ nine used self-reported questionnaires^19 27 31 32 41 44 57 60 65^ and the rest did not specify how the questionnaires were administered^67^ (online supplemental file 2).

Some studies used researcher-developed questionnaires based on themes identified in the literature, which were either pretested through pilot surveys or validated by experts, while others adapted questionnaires from previous studies.^1921 27 29^,^33 35^ (online supplemental table S2.2).

Several studies employed standardised tools such as the Patient Satisfaction Questionnaire III;^43^ the Patient Satisfaction Questionnaire;^20 44^ the Client Satisfaction Questionnaire scale;^42^ the Quality of Prenatal Care Questionnaire;^38^ the Modified Six Simple Questionnaire;^64^ the Hospital-based Intrapartum Care Scale;^34^ the Women’s views of Birth Labour Satisfaction Questionnaire; and the Gungor, Beji, Development and Psychometric Testing of the Scales.^41^

Across the studies, different methods were employed to measure satisfaction. These included score-based systems such as Likert-type scales ranging from 3 to 7 points.^19^,^2127 31^ Others used options like ‘yes/no’, ‘good/poor’ or ‘satisfied/not satisfied/neutral’.^2937 39 40 46^,^51 53^ Some studies included questions such as ‘Would you register again and/or recommend this facility?’ and ‘What was the reason for your satisfaction/dissatisfaction?’.^3046 47 50 51 55 56 65^,^67^

Qualitative research studies, on the other hand, used in-depth interviews and focus group discussions guided by literature to explore respondents’ experiences, satisfaction factors, reasons for dissatisfaction and recommendations for healthcare improvement.^62^,^65^ One study specifically focused on deaf individuals and used a semistructured video-recorded one-on-one sign language interview.^61^

### Satisfaction with care

MCS was reported as a proportion in 31 studies, which constituted the majority of the studies (online supplemental table S1.2). In each of these studies, satisfaction scores were extracted directly from the study based on responses to specific questions (online supplemental table S2.2). However, in six studies, scores were calculated by combining satisfaction rates from related subquestions or subgroups within the participants' responses.^19 44 54 57 64 66^ Of the 31 studies, 30 reported their findings in percentages, while one used median scores for satisfaction with delivery care. The median scores, ranging between 6 and 7 out of a maximum of 7, were converted to percentages, yielding a range of 86% to 100%.^64^ Most studies indicated high MCS rates in Nigeria, with 20 studies reporting rates of 80% and above. Ten studies showed satisfaction ranging between 79% and 40%, while one study reported satisfaction below 40% (see online supplemental table S1.2 for details).

### Determinants of maternal care satisfaction

**Figure 2:**
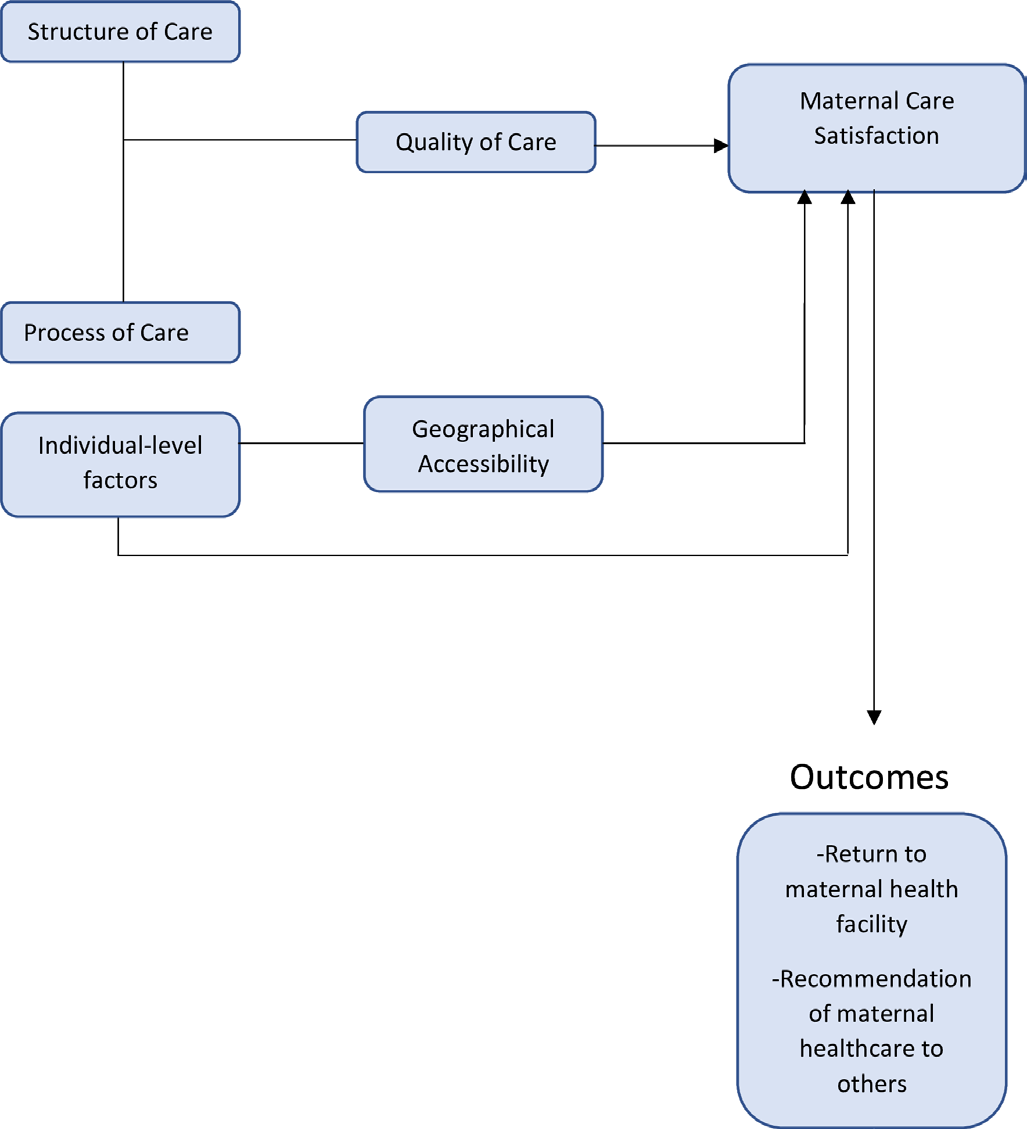
Conceptual framework of the determinants of maternal care satisfaction in Nigeria. This diagram illustrates the various factors associated with maternal care satisfaction. It emphasises that the structure and process of care significantly influence care quality, which, in turn, affects satisfaction. Individual-level factors and geographic accessibility are also associated with satisfaction. The outcomes of maternal care satisfaction include an increased likelihood of returning to health facilities and recommending care to others.

The factors influencing MCS were categorised into themes related to care structure, care process, geographic accessibility and individual-level factors (table 2 and figure 2).

**Table 2:** Determinants of satisfaction with maternal healthcare

Factors	Details	
	Satisfaction	Dissatisfaction
**Structure**		
Physical facilities	Clinic cleanliness, lighting, spacious rooms, low noise, registration process, opening hours, service quality and ward management.^3234 42 43 47^,^49 57^	Frequent strikes, dirty hospitals, water shortages, inadequate equipment, power disruptions and poor bed allocation.^30 62 63 65^
Human resources		Inadequate staffing and inconsistency in seeing the same doctor.^30 63 65^
Medical equipment and supplies	Adequate supplies, clean trays and equipment, and easy access to prescribed medication^34 43 48 49 57^	Poor radiological and laboratory services.^62^
**Process of care**		
Clinical effectiveness and safety	Effective communication, safety, privacy, politeness, respect, empathy, a positive interpersonal relationship with patients and involvement of patients’ partners in the maternal care process.^3436 37 42 46^,^48 50 53 58^	Rushed doctors, unfriendly providers, lack of privacy, pressure during labour, uninteresting health talks and poor attention during labour.^20 30 32 39 62 65 67^
Timeliness	Waiting time of less than 45 min.^29^	Long waiting times, delays at service points and late arrival to work by service providers.^21 30 32 39 42 46 47 53 59 60 62 63 65 67^
Cost-effectiveness	Reduced service costs^47 57^	High costs of health services and medical supplies.^29 32 42 59 65^
Equitable	Non-discriminatory treatment regardless of socioeconomic status.^48 50 53^	Discrimination and stereotyping among deaf women and language barriers.^61^
**Geographic accessibility**		
Proximity	Geographic accessibility, transportation and close proximity to the health facility.^32 43 47 65^	
Individual-level factors	Woman’s education, income, occupation, awareness of health facilities, age, gestational age, parity, religion, region, number of children, facility type, delivery mode, satisfaction with past experiences, day of discharge after an elective uncomplicated elective caesarean section, perceived quality of care and expectations.^2027 32 36 37 40^,^43 49 51 52 58 59 65 67^	

### Structure

The structure encompasses all elements that impact the context in which healthcare is delivered, such as physical facilities, human resources, and medical equipment and supplies. These factors include the following.

#### Physical facilities

Factors associated with satisfaction include cleanliness of the clinic environment, adequate lighting, spacious rooms, low noise, good registration process, adequacy of opening hours, satisfactory quality of services and effective management of the ward.^3234 42 43 47^,^49 57^ Likewise, women revealed that frequent health workers’ strikes, dirty hospital environments, inadequate water supply, lack of and insufficient equipment, irregular electricity and poor bed space allocation are causes of dissatisfaction with maternal health services.^28 62 63 65^

#### Human resources

Inadequate staffing in the health facility and the inability to see one doctor consistently were linked to dissatisfaction with maternal services among women.^28 63 65^ In a qualitative study conducted in Ogun State, it was revealed that unskilled health workers are the reason some women prefer to give birth in traditional birth centres instead of formal health facilities.^66^

#### Medical equipment and supplies

Adequate medical supplies, clean trays and equipment, the ability to find medical supplies when needed, an effective maternal care laboratory and getting prescribed medication easily were also linked to MCS.^34 43 48 49 57^ In Edo State, women stated that poor radiological and laboratory services are reasons for dissatisfaction with maternal services at referral hospitals.^62^

#### Process of care

The process of care indicates all the activities done by the care provider to improve the health of the people. We categorised the process of care into the following domains:

#### Clinical effectiveness and safety

Sixteen studies reported that carrying out necessary procedures or examinations on patients; meeting women’s health needs effectively; providers having a good attitude; being an empathic, caring, compassionate, and approachable health worker; safety during obstetric sonography; having midwives who are encouraging and reassuring; effectively communicating health conditions to patients; health workers having a healthy interpersonal relationship with clients; providers using polite language; ensuring privacy and confidentiality; and involvement of patients’ partners in the maternal care process are determining factors associated with increased satisfaction among women.^3234 36 37 42 46^,^48 50 53 57^ Women revealed that doctors being in a hurry, having unfriendly health providers, not providing privacy and confidentiality, pressuring women to have their babies quickly, and attending boring health talks and providers paying poor attention to women in labour are reasons for dissatisfaction with maternity care.^20 30 32 39 62 65 67^

#### Timelines

Long waiting times to receive treatment, delays at various service points in the facilities and late arrival to work by service providers are linked with dissatisfaction among women.^20 29 30 32 39 42 46 47 53 59 60 62 63 65 67^

#### Cost-effectiveness

Women are more likely to be satisfied with care when the cost of services is reduced.^47 57^ The high cost of health services and medical supplies are reasons for dissatisfaction with maternal care in Nigeria.^20 29 32 42 59 65^

#### Equitable

Providing non-discriminatory treatment regardless of patients’ socioeconomic status is associated with maternal satisfaction.^48 50 53^ In a qualitative study among deaf pregnant women attendingANC in Oyo, State Nigeria, it was revealed that women who attend publicly owned health facilities are more dissatisfied with care compared with those who attend privately owned facilities.^61^ Deaf women are usually discriminated against and stereotyped during antenatal training sessions due to their physical disability.^61^ In another study conducted in Lagos State, it was revealed that the language barrier is a challenge faced by pregnant women attending antenatal clinics.^32^

#### Geographical accessibility

We also found that geographical accessibility, transportation and distance to the health facility are associated with satisfaction.^32 47 65^ A study conducted in North-eastern Nigeria revealed that having easy access to tertiary hospitals is significantly associated with satisfaction with ANC services.^43^

#### Individual-level factors

Our study identified women’s educational status as a key individual-level factor associated with satisfaction with maternal care in Nigeria.^20 32 43^ Specifically, women with higher educational levels reported greater satisfaction with maternal healthcare services compared with their counterparts with lower educational status.^40 41 59^ Women with secondary or higher education are more likely to be satisfied with ANC than those with lower educational attainment.^37^ Interestingly, in Maiduguri, Borno State, clients with primary and secondary education expressed higher satisfaction levels than those with tertiary education.^43^ In contrast, some studies indicated no significant association between maternal education and satisfaction with perinatal care.^38 46 49 51 58 65^

We also found mixed results on the association between satisfaction with maternal care and women’s employment status or income level. While some studies reported an association,^20 32 43 51^ others found no significant association between satisfaction with pregnancy care and employment status or women’s monthly earnings.^38 42 46 49 51 58^

Additionally, we found that demographic factors such as parity and gestational age are associated with satisfaction with maternal health services in Nigeria. Women with two or more children are more likely to be satisfied with ANC than those with lower parity.^51^ A study in Saki, Oyo State, showed that being in the second trimester was associated with greater satisfaction with ANC.^44^ In contrast, studies conducted in Cross River, Ogun, Benin, Borno and Ibadan States found no significant association between parity or pregnancy status—whether primigravida or multigravida—and MCS.^38 42 43 46 49 59^

Other individual-level factors associated with MCS in Nigeria include being over 25 years of age, awareness of the existence of a health facility, marital status, belonging to the Christian faith, type of facility, mode of delivery (vaginal birth or caesarean section), elective versus emergency caesarean delivery, day of discharge from the hospital, satisfaction with previous pregnancy or delivery experiences at the facility, perceived quality of care, and level of expectations^1930 32 36 37 40^,^43 49 51 52 58 59 65 67^

In summary, key factors influencing MCS in Nigeria include positive client-provider relationships, a supportive and accessible hospital environment, equitable care centred around patient’s needs and affordable service costs. A summary of these determinants is provided in table 2.

### Outcome

Satisfaction with maternal healthcare is associated with future health-seeking behaviour and willingness to recommend care. Women reported that they would return to the same health facility for future pregnancies and recommend it to others.^2138 41 42 45^,^47 50 55 56 59 63^ Respondents in a focus group in Ogun State reported that women had negative experiences with antenatal and postnatal care due to hostile attitudes from health workers, causing them and their friends to avoid returning to the health facility.^66^ Some women said they would go to traditional birth places instead for subsequent births.^66^

## Discussion

### Maternity care satisfaction

Patient satisfaction has become an essential part of the healthcare system globally. Our study shows that maternal health services, including antenatal, intrapartum and postnatal care, have received high patient satisfaction scores across different settings in Nigeria, with most of the articles reviewed scoring between 80% and 98.5%. This is comparable to the findings from maternity satisfaction studies conducted in other low- and middle-income countries where high MCS scores were also reported.^68^,^72^

### Factors influencing the measurement of maternal care satisfaction (MCS

Evidence shows that high MCS scores are reported not only in LICs but also in HICs, where there is better infrastructure and higher-quality health systems.^73^,^75^ This raises uncertainty about whether the high satisfaction scores in low-resource settings such as Nigeria accurately represent the mothers’ experiences or are influenced by measurement or reporting factors.^76^

While the high satisfaction rates reported in this study may reflect genuine positive experiences of women despite a struggling maternal health system, it is also important to consider other factors that might influence these results. One is response bias which occurs when respondents answer survey questions in ways that do not accurately reflect their true responses.^77^ Social desirability bias, for instance, may arise when respondents know that their answers reflect negatively or positively on themselves.^77^ Our findings show that most satisfaction surveys in this review used interviewer-administered questionnaires, which may compromise the confidentiality and honesty of responses. Additionally, respondents may feel the need to please health providers and alter their answers based on how it could affect their future care.

Extreme response bias is another type of response bias which occurs when a respondent gives an extreme answer even if they do not hold an extreme opinion. This is common in satisfaction surveys,^78^ particularly with Likert scale questions, which is the most frequently used questionnaire design method, reported in our study. Low education status or poor understanding of survey questions may create extremely biased responses.

An additional response bias that could impact survey results is agreement bias.^78^ It happens when a respondent repeatedly answers positively to every question without much thought. This could occur if survey questions are excessively long, causing fatigue. It could also occur when questions are biased toward getting positive feedback. For instance, if a woman is asked to rate her level of agreement or disagreement with a statement like ‘I was satisfied with the friendliness of the staff’, she may choose ‘agree’ without considering her ‘true’ preference, simply because she believes that is the expected response. To avoid agreement bias, researchers should avoid leading questions and keep questionnaires short to reduce survey fatigue.

#### Process and Outcome

Aside from response bias, satisfaction measures may be influenced by the difficulty distinguishing the care process from health outcomes.^79^ For example, women in countries such as Nigeria, where maternal and neonatal mortality rates are high, may give high satisfaction ratings after childbirth, even if they were mistreated in the hospital. This is due to the joy of a live birth, which can overshadow any negative experiences during the delivery. While the newborn’s condition at birth is indeed a critical indicator of care quality, it should not overshadow the assessment of the care process itself, as both aspects are equally indicative of care quality. This is evident in a study in Zambia, where it was revealed that a baby’s condition at birth is associated with women’s satisfaction with immediate postnatal care.^80^ To accurately measure satisfaction ratings, researchers should clearly differentiate between healthcare processes and outcomes. Additionally, open-ended questions and qualitative research methods may be more effective than Likert-scale questionnaires in capturing patient experiences with maternal care. Moreover, future researchers should also be mindful of the possibility that the most satisfied women may be more likely to respond to satisfaction surveys, which could inflate satisfaction rates.^81^ This is particularly important when conducting surveys after delivery, as the immediate postnatal period can significantly influence women’s perception of care. To address this potential bias, researchers should actively strive for balanced participation. This can be achieved by reaching out to women with various childbirth experiences—both positive and negative. Efforts should also be made to follow-up with non-respondents to help reflect a broader range of perspectives and ensure more accurate satisfaction data.

The goal of satisfaction surveys is to identify valuable aspects of the healthcare system that need improvement and hold healthcare providers accountable. If measurement errors occur, it becomes difficult to improve health quality. Future research should, therefore, consider the suggestions raised in this study when designing survey tools to reflect women’s lived experiences accurately.

#### Balancing genuine satisfaction and bias

Despite the possibility of measurement and response biases, it is important to acknowledge that high satisfaction scores could still reflect the actual experiences of women. Satisfaction is influenced by a complex interplay of personal experiences and expectations of patients, which could result in high scores despite deficiencies in service quality.^32 43 50^ Women may feel genuinely satisfied even in low-resource settings, particularly when their expectation is met or exceeded.

The relationship between personal experiences and expectations highlights the inherent subjectivity of satisfaction surveys.^11 82^ While some may view this subjectivity as a limitation that raises concerns about bias or variability, it is precisely this aspect that makes satisfaction surveys essential for evaluating and improving healthcare delivery.^82^ Patients come from diverse backgrounds and have different needs and expectations. By including their subjective views, we can gain a more comprehensive understanding of these needs and ensure that tailored care is provided and creating a responsive and effective maternal health system. Therefore, future researchers should prioritise designing satisfaction surveys that not only quantify women’s experiences but also capture the details of their expectations.

### Determinants of satisfaction with maternity care

MCS is influenced by several factors. Good client-provider relationship is linked to patient satisfaction with maternity care in Nigeria. A study in Italy similarly found that effective communication, listening to women’s needs, involvement, and respectful and timely care positively influenced satisfaction levels.^73^ Negative behaviour of health providers during maternity care in Mozambique led to dissatisfaction among women, while care and positive attitude from professionals in Ghana increased the satisfaction of participants.^69^

Waiting time significantly impacts MCS. A review from Ethiopia found that women who waited for 15 min or less to see a health provider were 3.66 times more likely to be satisfied with delivery services than those who waited for more than 15 min.^83^ Better interpersonal relationships between healthcare providers and patients are crucial for improving maternal care in Nigeria.

Hospital environment and accessibility are important factors that influence MCS satisfaction in Nigeria. Women prefer health facilities that are affordable, well-equipped and located near them.^12 73^ Cost of care positively affects MCS in Nigeria, as shown in our study. Similar findings were reported in Northwest Ethiopia and Nepal, where cost-free pregnancy care services were associated with satisfaction with maternal care services.^17 68^ Women in Nigeria may avoid giving birth in hospitals due to the cost of transportation and accommodation.^32^ This, along with high poverty rates, makes it difficult for them to access formal health services. Implementing health insurance schemes, especially for the economically disadvantaged, could ensure that pregnant women receive quality care, regardless of income or location.

Furthermore, we found that the correlation between MCS and women’s socioeconomic factors, such as education, occupation and income level, yielded inconsistent findings. The mixed results may be due to differences in study settings, research methodology or socioeconomic contexts. A similar study in Zambia reported that apart from employment status, there is no association between women’s background characteristics and satisfaction levels.^80^ In another study in Nepal, there was no association between women’s socioeconomic and demographic factors and MCS.^68^

Additionally, our study demonstrated a correlation between MCS and demographic factors. However, there was no complete consensus within the literature about this correlation. We observed significant associations between MCS and women’s age, gestational age and parity, with higher satisfaction levels linked to increasing age and parity. This finding is consistent with studies conducted in Italy and Norway, where multiparous and older women, respectively, were found to be more satisfied with postnatal care.^76 77^ One possible explanation for this phenomenon is that older and multiparous women often possess prior experience in caring for newborns, which may lead to reduced anxiety levels and, consequently, higher satisfaction with MCS. Another possible reason for this is selection bias. Women who had a negative experience with maternal healthcare may be less likely to return to the same facility for subsequent childbirth, thus not participating in satisfaction surveys for later delivery. This could thereby lead to an overrepresentation of women with higher parity reporting high satisfaction. However, the lack of consensus on these correlations suggests that MCS may also be shaped by other factors, such as quality of care, resource availability, and individual expectations, which can differ across settings.

### Outcome of satisfaction

Satisfaction with maternal healthcare in Nigeria is closely linked to women’s intentions to seek continued care and recommend services to others. A study conducted across secondary and tertiary health facilities in Ibadan revealed that 93.5% of satisfied women expressed a desire to return to the health facility for subsequent maternal healthcare, and 97.3% indicated they would recommend the facility to friends and family members.^38^ Research in eastern Nigeria further demonstrated that satisfaction with ANC significantly increases the likelihood of facility-based deliveries, emphasising the importance of ensuring patient satisfaction.^55^

These patterns are not unique to Nigeria; studies in Nepal and Mozambique have shown that satisfied women are more likely to express intentions to return for future deliveries and to recommend services to others.^68 70^ For instance, nearly half of women in eastern Ethiopia reported delivering at health facilities based on recommendations, highlighting how patient satisfaction impacts return to the health facility.^84^ Likewise, a study among postpartum women across four developing countries, including Nigeria, found that women who did not experience mistreatment—such as verbal or physical abuse—were significantly more likely to report higher satisfaction with care and recommend the facility to others.^64^ Specifically, women who were not verbally abused were 4.4 times more likely to recommend the facility compared with those who experienced such mistreatment.^64^ Additionally, shorter waiting times were associated with a higher likelihood of recommending the facility, further emphasising how patient satisfaction influences maternal healthcare utilisation.^64^

Similarly, the experiences shared by women in a focus group discussion in Ogun State, Nigeria, illustrate the negative impact of dissatisfaction.^66^ Respondents reported that hostile attitudes from health workers discouraged them and their friends from returning to health facilities, with some indicating a preference for traditional birthplaces for future deliveries.^66^ This highlights the importance of healthcare providers creating a welcoming and respectful environment. Positive patient interactions can significantly enhance satisfaction, which is essential for improving continued service utilisation and health outcomes in the community.

### Strengths and limitations

This systematic review study investigated MCS in Nigeria and has some notable strengths. First, it covers all domains of maternal health services across a broad range of databases. Second, we excluded studies where women were pregnant more than 1 year ago to reduce recall bias. Additionally, our study assessed the measurement of MCS in Nigeria, a dimension not explored in previous satisfaction studies. Finally, the study examines the impact of maternity satisfaction on continued care and service recommendations. A limitation of the study is that a meta-analysis could not be conducted, making it difficult to draw precise, quantifiable conclusions. Additionally, most of the included studies were cross-sectional, limiting causal inference. We cannot exclude the issues around measurement bias discussed above.

### Implications for practice, policy and future research

The findings from this study can guide healthcare practitioners in developing targeted interventions to improve maternal care quality in Nigeria and similar contexts. Additionally, it provides a policy roadmap for enhancing pregnancy outcomes and patient satisfaction. Future research in this area could explore longitudinal patient journey study designs and employ meta-analytic approaches for a more robust understanding of MCS and its determinants. High MCS ratings in Nigeria may show genuine positive experiences, but they could also be shaped by measurement-related biases, emphasising the importance of improving research methods to reflect women’s experiences and expectations accurately.

## Conclusions

This study found that while satisfaction with maternal services in Nigeria is high, it may indicate genuine positive experiences or be influenced by other factors such as measurement-related biases. Satisfaction with the quality of perinatal care is fundamental to establishing standards for maternal health services and reducing maternal morbidity and mortality. When assessing maternal satisfaction, it is important for health researchers to carefully design data collection tools to ensure that women’s reported satisfaction ratings accurately reflect their lived experiences. Furthermore, this study highlights the multifaceted nature of factors influencing maternal satisfaction and spanning aspects such as the structural and procedural facets of care, geographical differences and individual-level characteristics. Therefore, healthcare delivery interventions should be tailored to the local needs of women to enhance pregnancy outcomes.

## Supplementary material

10.1136/bmjph-2024-001278 online supplemental file 1

10.1136/bmjph-2024-001278 online supplemental file 2

## Data Availability

All data relevant to the study are included in the article or uploaded as supplementary information.
