# Supplementary material for: Measurement, determinants and outcomes of maternal care satisfaction in Nigeria: a systematic review
Source: BMJ Public Health. 2025 Feb 11;3(1):e001278. doi: 10.1136/bmjph-2024-001278 (PMC11843491; doi:10.1136/bmjph-2024-001278)
Supplement: online supplemental file 1 [file bmjph-3-1-s001.pdf]

Supplementary Table S1.1: Complete Search Strategy

S/N	Search String
1	exp Mothers/ or exp Patients/ or exp Women/ or exp Female/
2	exp Patient Satisfaction/
3	exp Personal Satisfaction/
4	2 or 3
5	1 and 4
6	((client$ or women or woman or mother$ or patient$) adj3 (satisf$ or experience$ or respect$ or digni$ or quality or perception$ or ideation or perspect$ or Patient Reported Outcome$ or view or views or expectation$ or perceive$ or belief$ or believe$ or perception$ or attitude$ or perspective$ or opinion$ or think$)).mp.
7	5 or 6
8	exp Pregnant Women/ or exp Pregnancy/ or exp Infant, Newborn/ or exp Pregnancy/ or exp Delivery, Obstetric/ or exp Midwifery/ or exp Breast Feeding/ or exp Maternal Health Services/ or exp Hospitals, Maternity/ or exp Prenatal Care/
9	(pregnan$ or matern$ or antenatal or intrapartum or postnatal or post-natal or birth$ or Childbirth$ or labor or labors or labour$ or caesarean section$ or c section$ or abdominal deliver$ or postcaesarean section$ or obstetric$ or midwife$ or Breast feed$ or neonat* or newborn* or new born*).mp.
10	8 and 9
11	Nigeria.mp. or exp Nigeria/
12	7 and 10 and 10

Supplementary Table S1.2: Characteristics of the studies included in the systematic review of patient satisfaction with health services in Nigeria

S/N	Author	Year	State	Maternity services received	Facility type	Research type	Study Design	Research instrument/tool	Sample size	Proportion who were satisfied	Quality assessment (%)
**1**	Abdus-salam et al.[29]	2021	Ibadan	Antenatal	Tertiary	Quantitative	Cross-sectional	Questionnaire	122	68.90%	61.5
**2**	Addah et al [30]	2016	Bayelsa	Antenatal	Tertiary	Quantitative	Cross-sectional	Questionnaire	198	-	56.41
**3**	Ademuyiwa et al. [27]	2021	Lagos	Antenatal	Secondary	Quantitative	Cross-sectional	Questionnaire	1316	-	61.54
**4**	Ademuyiwa et al. [31]	2020	Lagos	Antenatal	Secondary	Quantitative	Cross-sectional	Questionnaire	1316	-	71.79
**5**	Ademuyiwa et al. [32]	2020	Lagos	Antenatal	Secondary	Quantitative	Cross-sectional	Questionnaire	1316	-	58.97
**6**	Adigun et al [61]	2020	Oyo, Ibadan	Antenatal	Unspecified	Qualitative	Cross-sectional	Semi-structured interview guide	9	-	74.36
**7**	Agbata et al.[33]	2018	Ebonyi	Antenatal	Tertiary	Quantitative	Cross-sectional	Semi-structured questionnaire	335	71.50%	51.28
**8**	Alabi et al. [19]	2023	Ibadan	Antenatal	Tertiary	Quantitative	Cross-sectional	Questionnaire	289	94.80%	53.85
**9**	Ali et al. [34]	2022	Jos	Delivery	Tertiary	Quantitative	Cross-sectional	Questionnaire	173	86.70%	61.54
**10**	Anikwe et al [35]	2019	Ebonyi	Delivery	Tertiary	Quantitative	Cross-sectional	Questionnaire	243	59.3%	58.97
**11**	Anikwe et al [36]	2020	Ebonyi	Antenatal	Tertiary	Quantitative	Cross-sectional	Questionnaire	284	89.40%	69.23
**12**	Asekun-Olarinmoye et al.[67]	2009	Osun	Antenatal	Tertiary	Quantitative	Cross-sectional	Questionnaire	289	77.50%	61.54
**13**	Bako et al. [37]	2022	Nasarawa	Antenatal	Primary	Quantitative	Cross-sectional	Questionnaire	450	29.30%	69.23
**14**	Banke-Thomas et al [64]	2017	Lagos	Delivery	Secondary and tertiary facility	Mixed-methods	Cross-sectional	Focus group discussion guide (qualitative research) and Questionnaire (quantitative research)	1000 (quantitative) and 39 (qualitative)	Median score (6-7 out of 7) 85%-100%	64.10
**15**	Bello O. [38]	2018	Ibadan	Antenatal	Secondary and tertiary facility	Quantitative	Cross-sectional	Questionnaire	500	98.40%	64.10
**16**	Ekott et al [39]	2013	Port-harcourt	Antenatal	Primary	Quantitative	Cross-sectional	Questionnaire	400	94.00%	48.72
**17**	Enabudoso et al. [40]	2011	Edo	Delivery	Tertiary	Quantitative	Cross-sectional	Questionnaire	211	80%	71.79
**18**	Esan et al. [41]	2022	Ekiti	Delivery	Primary and tertiary	Quantitative	Cross-sectional	Questionnaire	267	94.80%	61.54
**19**	George et al. [42]	2022	Cross River	Antenatal	Tertiary	Quantitative	Cross-sectional	Questionnaire	200	92.00%	71.79
**20**	Ibrahim et al. [43]	2017	Maiduguri, Borno	Antenatal	Tertiary	Quantitative	Cross-sectional	Questionnaire	274	98.20%	64.10
**21**	Ishola and Kazeem [44]	2022	Saki-west, Oyo	Antenatal	Primary	Quantitative	Cross-sectional	Questionnaire	582	13.40%	51.28
**22**	Maung et al. [45]	2020	Nigeria	Delivery	Secondary and tertiary facility	Quantitative	Cross-sectional	Questionnaire	507	85.00%	79.49
**23**	Nwaeze et al. [46]	2013	Ibadan	Antenatal	Tertiary	Quantitative	Cross-sectional	Questionnaire	239	81.10%	51.28
**24**	Obagha et al [20]	2020	Anambra	Antenatal	Primary and secondary	Quantitative	Cross-sectional	Questionnaire	284	62.20%	66.67

**25**	Odetola and Fakorede [65]	2018	Ibadan	All three	Primary	Mixed-methods	Cross-sectional	Questionnaire	66	98.50%	61.54
**26**	Okedo-Alex et al. [47]	2021	Ebonyi	Delivery	Tertiary	Quantitative	Cross-sectional	Questionnaire	574	-	66.67
**27**	Okonofua et al. [48]	2020	Niger, Benin, Delta, Abuja	All three	Secondary	Quantitative	Quasi-experimental	Questionnaire	2262	-	74.36
**28**	Okonofua et al. [62]	2017	Kano, Kaduna, Niger, Abuja, Oyo, Ogun, Delta, Edo.	All three	Secondary and tertiary facility	Qualitative	Unspecified	FGD guide	NA	-	71.79
**29**	Okoror et al. [49]	2020	Edo	Antenatal	Primary	Quantitative	Cross-sectional	Questionnaire	405	83.2%	53.85
**30**	Olabode et al. [63]	2022	Kwara	Delivery	Secondary	Qualitative	Cross-sectional	IDI guide	16	-	69.23
**31**	Oladapo et al. [50]	2008	Ogun	Antenatal	Primary	Quantitative	Cross-sectional	Questionnaire	452	81.4%	66.67
**32**	Oladapo et al. [51]	2009	Ogun	Antenatal	Primary	Quantitative	Cross-sectional	Questionnaire	452	-	71.79
**33**	Onu et al. [52]	2021	Abakaliki	Delivery	Tertiary	Quantitative	Randomize controlled trial	Questionnaire	228	100% (day-2 discharge); and 76.8% (day-5-7 discharge)	74.36
**34**	Onyeajam et al. [53]	2018	Adamawa, Nasarawa, Benue and Taraba	Antenatal	Primary and secondary	Quantitative	Cross-sectional	Questionnaire	1336	-	79.49
**35**	Orhue et al. [54]	2020	Edo	Delivery	Tertiary	Quantitative	Randomize controlled trial	"WHO modified partograph, and interview	320	88.15%	71.79
**36**	Ossai et al. [55]	2020	Ebonyi	Antenatal	Tertiary	Quantitative	Cross-sectional	Questionnaire	415	73%	69.23
**37**	Ossai et al. [56]	2015	Enugu	All three	Primary	Quantitative	Cross-sectional	"Questionnaire (and the National Primary Health Care Development Agency (NPHCDA) for equipment and personal tool)	540	70.2%	84.62
**38**	Oyediran et al. [57]	2022	Lagos	Delivery	Tertiary	Quantitative	Cross-sectional	Questionnaire	259	59.10%	38.46
**39**	Peace et al. [58]	2022	Ibadan	Delivery	Mixed	Quantitative	Cross-sectional	Questionnaire	270	62.9%	58.97
**40**	Sodeinde et al. [59]	2020	Ogun	Antenatal	Primary	Quantitative	Cross-sectional	Questionnaire	380	96.8%	64.10
**41**	Sufiyan et al. [21]	2013	Kaduna	Antenatal	Primary	Quantitative	Cross-sectional	Questionnaire	234	74%	51.28
**42**	Udoh et al. [60]	2011	Southeastern Nigeria	Antenatal	Mixed	Quantitative	Cross-sectional	Questionnaire	648	-	56.41
**43**	Umahi et al. [66]	2019	Ogun	Antenatal and Postnatal	Primary	Mixed methods	Cross-sectional	Questionnaire	389	83%	69.23
